# A 3-year application of different mycorrhiza-based plant biostimulants distinctively modulates photosynthetic performance, leaf metabolism, and fruit quality in grapes (*Vitis vinifera* L.)

**DOI:** 10.3389/fpls.2023.1236199

**Published:** 2023-08-29

**Authors:** Paola Ganugi, Tito Caffi, Mario Gabrielli, Elena Secomandi, Andrea Fiorini, Leilei Zhang, Gabriele Bellotti, Edoardo Puglisi, Monica Broussard Fittipaldi, Florencia Asinari, Vincenzo Tabaglio, Marco Trevisan, Luigi Lucini

**Affiliations:** ^1^ Department for Sustainable Food Process, Università Cattolica del Sacro Cuore, Piacenza, Italy; ^2^ Department of Sustainable Crop Production, Università Cattolica del Sacro Cuore, Piacenza, Italy; ^3^ Department of Sciences, Technologies and Society, University School for Advanced Studies, IUSS, Pavia, Italy

**Keywords:** fruit quality, metabolomics, multivariate analysis, photosynthetic parameters, plant symbiont, arbuscular mycorrhizal fungi

## Abstract

The use of microbial biostimulants in agriculture is recognized as a sustainable approach to promoting crop productivity and quality due to improved nutrient uptake, enhanced stress tolerance, and improved ability to cope with non-optimal environments. The present paper aimed to comparatively investigate the effect of seven different commercial mycorrhizal-based treatments in terms of yield, phytochemical components, and technological traits of Malvasia di Candia Aromatica grape (*Vitis vinifera* L.) plants. Metabolomic analysis and photosynthetic performance were first investigated in leaves to point out biochemical differences related to plant growth. Higher photosynthetic efficiency and better PSII functioning were found in biostimulant-treated vines, reflecting an overall decrease in photoinhibition compared to untreated plants. Untargeted metabolomics followed by multivariate statistics highlighted a robust reprogramming of primary (lipids) and secondary (alkaloids and terpenoids) metabolites in treated plants. The analysis of berry yield and chemical components exhibited significant differences depending on the biostimulant product. Generally, berries obtained from treated plants displayed improved contents of polyphenols and sugars, while yield remained unchanged. These results elucidated the significant role of microbial biostimulants in determining the quality of grape berries and eliciting biochemical changes in vines.

## 1 Introduction

Despite predictions showing an increase in agricultural production by 2050, agricultural output will not be sufficient under exacerbated climate change (CC) conditions, natural resource degradation, and biodiversity losses (Calicioglu et al., 2019). In this scenario, biostimulants stand out as one of the most promising options to achieve low-input agriculture within a sustainable food system by ensuring efficient management of natural resources, securing yield and quality of the production, and improving plant tolerance to abiotic stresses (Rouphael and Colla, 2020). Defined by the European Parliament and the Council in Regulation 2019/1009, biostimulants stimulate nutrient use efficiency, tolerance to abiotic stresses, quality traits, and availability of confined nutrients in soil or rhizosphere. Among the category, microbial biostimulants represent a sub-group that includes microorganisms like arbuscular mycorrhizal fungi (AMF) and N-fixing bacteria belonging to the *Azotobacter*, *Rhizobium*, and *Azospirillum* genera. The use of single microorganisms or consortia has been demonstrated to increase crop productivity and quality through enhanced nutrient uptake and abiotic stress tolerance, even though the specific climatic conditions, plant species, and soil are essential factors to be considered (Backer et al., 2018; Bitterlich et al., 2018; Turrini et al., 2018; Woo and Pepe, 2018). Notwithstanding, the advantage of AMF application may embed systemic resistance induction, endowing plants with disease and pest attack protection (Cameron et al., 2013).

As the effect of CC is substantial across all crops, it is striking for specialty crops like wine grapes (Fraga et al., 2016). Altered precipitation patterns leading to drought periods, increase in temperature, and frequency of extreme weather events have adverse effects on grapevine growth, berry quality, and yield (Kuhn et al., 2014; Fraga et al., 2016; Torres et al., 2018b; Fraga et al., 2020). Specifically, heat stress results in a modification of phenology timing with the hastening of ripening of grapes and lower quality of berries due to reduced sugar content, titratable acidity, and an altered concentration of secondary metabolites with emphasis on polyphenols (Mori et al., 2007; Sadras and Moran, 2013; Arrizabalaga et al., 2018; Torres et al., 2018c; Arrizabalaga-Arriazu et al., 2020). Despite requiring relatively low water inputs, grapevine impacted by water shortages results in lowered berry size and decreased malic acid concentration (Matthews and Anderson, 1988; Bondada and Shutthanandan, 2012; Gambetta, 2016). While having a positive effect on vineyard microbial networks and, consequently, on soil quality (Torres et al., 2021) and possibly facilitating the further colonization by other resident AMF taxa (Nogales et al., 2021), AMF have also an impact on the overall plant performance, including agronomical traits and biochemical processes (Trouvelot et al., 2015; Ganugi et al., 2019). Moreover, the application of AMF can mitigate abiotic stresses, including high temperatures, UV-B radiation, and drought stress (Nikolaou et al., 2003; Torres et al., 2018a; Nogales et al., 2021). However, it is difficult to generalize the outcomes of AMF application since different responses have been reported, likely depending on grape genotype and fungal species applied (Linderman and Davis, 2001; Aguín et al., 2004; Krishna et al., 2005; Bruisson et al., 2016; Torres et al., 2016; Torres et al., 2018b). In addition to the effects on root volume, length, and morphology, AMF inoculation can increase the number of leaves per vine, leaf area, and total dry weight (Aguín et al., 2004; Derbew et al., 2007). Indeed, the effect of mycorrhizal inoculation induces a shift in plant photosynthetic performance and relative water content (RWC), with a positive impact on physiological and nutritional status (Krishna et al., 2005). Moreover, by modulating the biochemical processes, AMF biostimulants influence the hormone metabolism, increase chlorophyll and carotenoids, and promote the accumulation of N, P, Mg, and Fe (Gabriele et al., 2016; Torres et al., 2018a; Torres et al., 2018b; Antolín et al., 2020; Karoglan et al., 2021). The possible effects of AMF on yield and nutritional parameters have been given great importance. Mycorrhizal inoculation leads to an increase in yield, cluster number per vine, and cluster weight, thus reducing the berry weight (Antolín et al., 2020; Karoglan et al., 2021). The modulation of the phytochemical composition and antioxidant properties has been also investigated, demonstrating a variation in polyphenols like anthocyanin, flavanols, phenolic acids, and flavan-3-ols as well in soluble solids following AMF inoculation (Gabriele et al., 2016; Torres et al., 2018b; Torres et al., 2018c; Antolín et al., 2020; Karoglan et al., 2021).

Nonetheless, the cultivar and microbial strain or consortia applied are of crucial importance in framing applicative results in crops coming from AMF inoculation (Ganugi et al., 2021b).

Therefore, this study aimed to comparatively investigate the effect of seven different mycorrhizal-based treatments on photosynthetic performance and qualitative and quantitative traits of grapevine (cultivar Malvasia di Candia Aromatica) leaves and berries.

## 2 Materials and methods

### 2.1 Experimental site, treatments, and plant management

The field experiment was conducted by the Università Cattolica del Sacro Cuore in the vineyard of the farm Res Uvae in Castell’Arquato (Piacenza, northern Italy, 44°51′26.0″N 9°51′23.8″E), and grapevines (*Vitis vinifera* L.) of the white variety Malvasia di Candia Aromatica, grafted onto Kober 5BB rootstocks, were used. Plants were planted in 2019, spaced 1.2 m within and 2.5 m between rows, and pruned using the Guyot training system.

The characteristics in the top 0–30-cm soil layer were as follows: sand 242 g/kg, silt 356 g/kg, clay 402 g/kg, pH H_2_O 7.5, organic matter 8.8 g/kg, total N 0.71 g/kg, available P (Olsen) 32.07 mg/kg, exchangeable Ca 3,819.64 g/kg, exchangeable Mg 546.91 meq/100 g, exchangeable K 168.16 mg/kg, exchangeable Na 0.27 meq/100 g, and cation exchange capacity 26.1 meq/100 g.

Standard vineyard floor management practices—detailed in **Table S1**—included phytosanitary treatments according to Integrated Pest Management (IPM) principles and supported by DSS (vite.net from Horta s.r.l.) to estimate the proper timing of interventions.

The experimental design consisted of thirty-one 85-m-long adjacent rows of grapevines arranged to test mycorrhizal-based treatments (listed in **Table 1**). In detail, seven different AMF treatments were used: Micoflow + Radimix advance (T1), MycoApply DR (T2), MycoUp (T3), Overground ST-Plus (T4), Team mix (T5), Tricoveg (T6), and Vici Rhyzoteam WG (T7). A control condition (Control) was also added to compare the no-treatment effect. All treatments included four rows, except for T7, which had only three. The treatment dosage and time of application (described in **Table S2**) followed the label instructions. The biostimulants were applied once per year for three consecutive years, starting in 2019.

**Table 1:** List of the eight commercial products tested on grapevines (*Vitis vinifera* L.) cultivar ‘Malvasia di Candia Aromatica’.

Commercial product	Manufacturer	Code	Mycorrhizal inoculum	
Micoflow	Filnova	T1	*Rhizophagus irregularis* (labeled as *Glomus intraradices*)	1%
Radimix advance			*Rhizophagus* ssp. (labeled as *Glomus* spp.)	0.005%
MycoApply DR	Sumitomo Chemical Italia	T2	*R. irregularis* *Claroideoglomus luteum*, *Claroideoglomus etunicatum* *Claroideoglomus claroideum*	1%
MycoUp	Biogard®	T3	*Rhizophagus iranicus* (labeled as *Glomus iranicum*) var. *tenuihypharum*	120 propagules/g
Overground ST-Plus	Overtis	T4	*Rhizoglomus* spp. (labeled as *Glomus* spp.)	0.004%
Team mix	Hello Nature®	T5	*R. irregularis* (labeled as *G. intraradices*) *Funneliformis mosseae* (labeled as *Glomus mosseae*)	500 spores/g
Tricoveg	Chemia S.p.a.	T6	*Rhizophagus* spp. (labeled as *Glomus* spp.)	0.2%
Vici Rhyzoteam WG	Koppert	T7	*Rhizophagus* spp. (labeled as *Glomus* spp.)	4%

Manufacturer and code, respectively, indicate the corresponding producer and legend used for the manuscript. Control (T1), corresponding to non-inoculated vines, was also included in the experimental design.

Weather data were collected from the Università Cattolica del Sacro Cuore weather station (PESSL®). The Datalogger collected real-time data for in-field measurement of temperature, rain, flow rate, leaf wetness, and relative humidity, among other parameters. The data for the experimental period were downloaded from a web user interface.

### 2.2 Assessment of biostimulants

In the same experimental vineyard, the study of fungal ecology has been carried out with periodic sampling of roots and soil of the different treatments. The samplings were carried out every February for 3 years to determine fungal colonization. The samples were sent to the Biome Makers laboratory (Valladolid, Spain) for molecular analysis. In order to characterize fungal microbial communities, the internal transcribed spacer (ITS) marker regions were selected, and libraries were prepared following the two-step PCR Illumina protocol using custom primers amplifying the ITS1 region described previously (Becares and Fernandez, 2017). Then, they were sequenced by an Illumina MiSeq device using pair-end sequencing (Illumina®, San Diego, CA, USA). These data were used to check if the application of the different biostimulants worked and was able to increase the abundance of AMF in the roots of inoculated plants. Bioinformatic processing of the reads obtained and taxonomic annotation were performed using the Vsearch function in UCHIME and SINTAX algorithms (Edgar, n.d.; Edgar et al., 2011; Rognes et al., 2016). The operational taxonomic unit (OTU) tables generated for each year were used to evaluate the relative abundance within the fungal community from the database of sequenced species using the *dplyr* R package (Yarberry, 2021). Once detected, their relative abundance was evaluated over the years for each treatment in comparison to the relative abundance of the same genus population detected in Control.

### 2.3 Photosynthetic parameters in plant leaves

The MultispeQ 2.0 device (PhotosynQ, East Lansing, MI, USA) was used for photosynthetic analysis of grapevines. Starting July 14, 2022, field sampling was conducted once a week. Four plants per treatment were randomly chosen, and a lateral shoot’s fourth and sixth fully expanded leaves were used for data acquisition. The analyzed parameters included the quantum yield of photochemical energy conversion in PSII (Phi2), the quantum yield of non-regulated non-photochemical energy loss in PSII (PhiNO), and the quantum yield of regulated non-photochemical energy loss in PSII (PhiNPQ), which were calculated by applying the equations derived by Kramer et al. (2004) as modified by Tietz et al. (2017). Accordingly, the three light-adapted parameters add up to 1 (Phi2 + PhiNPQ + PhiNO = 1) (Kramer et al., 2004).

### 2.4 Untargeted metabolomic analysis of leaves

On July 26, 2022, four replicates per each mycorrhizal treatment were collected and freeze-dried. Specifically, each replicate was represented by a pool of a side shoot’s fourth and sixth leaves. The collection date (BBCH = 83) was defined on the basis of photosynthetic data, choosing the day on which the differences between the different treatments were most marked. Samples were ground with mortar and pestle using liquid nitrogen, and the leaves from the same plant were pooled. Then, extraction was performed according to Buffagni et al. (2021). Briefly, 1 g of plant material was extracted in 10 mL of 80% (v/v) methanol acidified with 0.1% formic acid. Mechanical homogenization was performed using an Ultra-Turrax (Polytron PT, Stansstad, Switzerland); then, extracts were centrifuged (12,000 × *g*) for 10 min and filtered on 0.22-µm cellulose filters into glass vials. The same extraction procedure was replicated twice from two independent sub-samples from each replication.

Untargeted metabolomic analysis was performed using a 1290 ultra-high pressure liquid chromatographic system coupled with a G6550 quadrupole-time-of-flight mass spectrometry (UHPLC/QTOF-MS) from Agilent Technologies (Santa Clara, CA, USA) as previously reported (Buffagni et al., 2021). Reversed-phase chromatography was performed using an Agilent Poroshell 120 PFP column (100 mm × 2.1 mm i.d., 1.9-μm particle size) and a mobile phase of water and acetonitrile (6%–94% acetonitrile in 33 min) both acidified with formic acid (0.1% v/v). The mass analyzer operated in positive mode (ESI+), and the mass spectrometer worked in full scan mode (range 100–1,200 *m*/*z*, 35,000 full width at half maximum (FWHM) nominal resolution).

The MassHunter Profinder 10.0 software (Agilent Technologies) was used to analyze raw data for alignments and annotation by applying the “find-by-formula” algorithm. In detail, mass (with a 5-ppm tolerance) and retention time (0.05 min maximum shift) alignment were combined with monoisotopic mass, isotopic accurate spacing, and ratio for compound annotation using the database PlantCyc 15.5, same as Hawkins et al. (2021). A filter reduction was then applied to annotated features, retaining compounds detected in at least 75% of replications within at least one treatment. According to COSMOS Metabolomics Standard Initiative (Salek et al., 2015), a Level 2 confidence in annotation, corresponding to putatively annotated compounds, was achieved (Schymanski et al., 2014).

### 2.5 Polyphenol profiling of fruits at harvest

To profile phenolic compounds in grapes, five bunches from five different plants per treatment were randomly collected at maturity on August 25, 2022, and stored at −20°C until analysis. Fruits of the same treatment were homogenized together, and the resulting pool was used to select four samples (2 g). Each sample was extracted in 20 mL of 80% methanol with 0.1% formic acid, centrifuged, filtered, and analyzed as previously described for leaf metabolomics.

The Agilent Profinder 10.0 software was used for data processing, and annotation of MS compounds was accomplished according to the Phenol-Explorer 3.6 database (Rothwell et al., 2013), using the algorithm above and achieving the same COSMOS level of confidence in annotation.

Finally, a semi-quantification of the phenolic compounds annotated was carried out. Phenolic compounds were grouped into sub-classes according to Phenol-Explorer annotations, and then a representative compound for each sub-class was used for semi-quantification. To this aim, a calibration curve was prepared for each class representative, based on standards (>98% purity, from Extrasynthèse, Genay, France) solutions in methanol 80% (v/v) analyzed under the same MS conditions. Specifically, the representative standards chosen were as follows: cyanidin (for anthocyanin equivalents), catechin (flavanols), quercetin (flavonols), luteolin (flavones, flavanones, dihydrochalcones, and isoflavonoids), sesamin (lignans), resveratrol (stilbenes), ferulic acid (phenolic acids), and tyrosol (tyrosol and other low molecular weight polyphenols). The results were expressed as equivalents (E)/g dry weight (dw) starting from the cumulate abundance of each compound within a phenolic sub-class.

### 2.6 Chemical and yield components of grapes at harvest

Yield was determined at harvest (August 25). To this aim, 25 vines per treatment were randomly chosen, and all plant bunches were weighted together to calculate the average yield component (kg). Moreover, the weight of 50 randomly selected berries per treatment was used for the weight component (g).

Technological parameters were evaluated at harvest by randomly selecting three bunches of three different plants per treatment. Bunches were put in a plastic sample bag, squeezed to obtain juice, and filtered for analysis. The total soluble sugar (TSS; °Brix) was determined by a digital refractometer (HHTEC). Must pH and titratable acidity were measured with an automatic titrator (Crison Instruments, Barcelona, Spain). Acidity was titrated with 0.1 N of NaOH to a pH 7.0 endpoint and expressed as g/L of tartaric acid equivalents, according to International Organisation of Vine and Wine (OIV) standard methods (OIV, 2021). The concentration of l-(+) tartaric and l-(−) malic were assessed using Hyperlab analyzers (Steroglass S.r.l., Perugia, Italy) by enzymatic methods (Baiano et al., 2015).

### 2.7 Statistical analysis

Statistical analysis of photosynthetic data, phenolic profiles, and fruits’ qualitative traits was completed on RStudio software (4.2.1 version). One-way analysis of variance (ANOVA) combined with *post hoc* Tukey’s honestly significant difference (HSD) test (*p*-value ≤ 0.05) was carried out to highlight differences among treatments.

The software Mass Profiler Professional 15.1 (Agilent Technologies) was used to elaborate metabolomics data. The abundance of compounds was Log2 transformed, normalized at the 75th percentile, and baselined against the median in Control. Fold change (FC)-based unsupervised hierarchical cluster analysis (HCA) based on Ward’s linkage method and Euclidean distance was used to naively point out metabolomic patterns. Then, ANOVA (Benjamini multiple testing correction) was performed to establish statistically significant differences compared to Control (*p*-value ≤ 0.05). Starting from the MS dataset, multivariate data analysis was completed in SIMCA® 17 (Sartorius, Umeå, Sweden). Therein, metabolomics data were Pareto-scaled and log-transformed, and then supervised modeling was carried out by orthogonal projection to latent structures discriminant analysis (OPLS-DA). Calculation of R2Y (goodness-of-fit) and Q2Y (goodness-of-prediction) was performed, the model was validated through CV-ANOVA (considering a *p*-value ≤ 0.05), permutation testing was used to exclude overfitting, and Hotelling’s T1 distance was applied to investigate potential outliers. Thereafter, the variable importance in projection (VIP) was used to select discriminant compounds, using a score threshold of ≥1.2. Discriminant markers were finally uploaded into the Pathway Tool of PlantCyc 15.1.0 software to facilitate biochemical interpretations (Hawkins et al., 2021).

After that, the AMF-driven changes in qualitative and quantitative traits of fruits were jointly evaluated using multivariate analysis of variance (MANOVA), and the Pillai trace test was used to calculate the *p*-value. Subsequently, canonical discriminant analysis (CDA) was conducted to identify differences among treatments and understand the relationships among the variables measured within those groups.

## 3 Results

### 3.1 Evaluation of successful biostimulant colonization after treatments

The presence of mycorrhiza was determined from the data obtained by the sequencing analysis of root samples. An increase in the population of these arbuscular symbiotic fungi has been observed over the years (**Figure S1**). Despite the limitations of the approach used, our sequencing data supported the success of the biostimulant treatments over the 3 years. The data refer to general AMF colonization, not limited to the applied strains, to account for the ecological processes driving mycorrhiza community dynamics post-inoculation. In fact, it has been suggested that knowledge of the past and present soil AMF community in the target field, and the local adaptation are essential to investigate colonization processes (Basiru and Hijiri, 2022). The whole AMF population may better represent community dynamics, as observed by Bender et al. (2019) in AMF-inoculated fields with highly diverse indigenous AMF communities. This is even more relevant when trials are performed for a long period of time following inoculation (Paz et al., 2021). Irrespective of the dynamics of the AMF community, improving AMF diversity and functions by commercial inoculants can contribute to the sustainability of the agroecosystem (van der Heyde et al., 2017).

On this basis, the AMF community has been investigated through ITS sequencing. Under our experimental conditions, after 3 years of application using a commercial strain, the abundance of AMF was double in the treated samples compared to untreated plants (Control).

### 3.2 Weather conditions and chlorophyll

**Figure 1:**
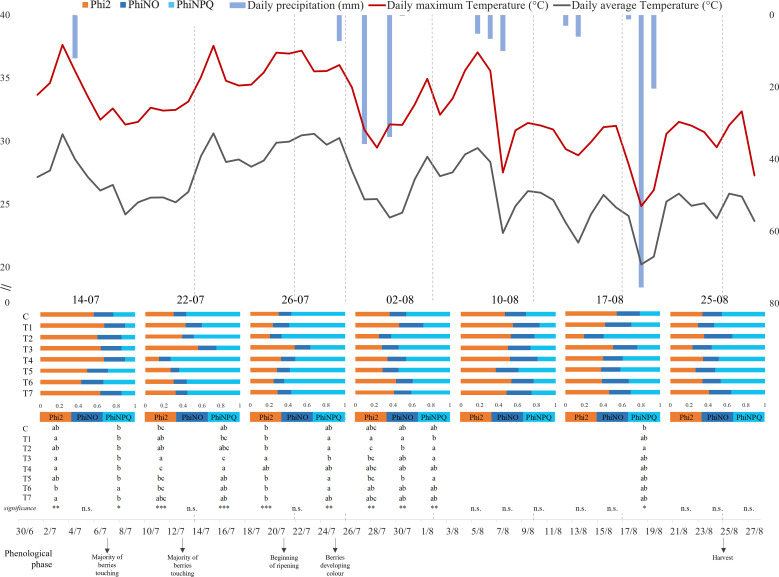
Graphical representation of climate trend and grapevine chlorophyll fluorescence parameters during the trial, between June 30 and August 27, 2022. The maximum and average daily temperature (°C) are represented by the red and gray lines respectively, while the daily precipitation (mm) is illustrated by the inverted blue histograms. Each bar represents mean values (n = 8) of energy partition for each treatment for each day of measurement. The bars represent the three complementary PSII quantum yield contributions: orange = quantum yield of photochemical energy conversion in PSII (Phi2), blue = quantum yield of non-regulated non-photochemical energy loss in PSII (PhiNO), and light blue = quantum yield of regulated non-photochemical energy loss in PSII (PhiNPQ). Letters indicate homogenous sub-classes resulting from ANOVA (*p*-value ≤ 0.05, Tukey’s *post hoc* test). *, p-value < 0,05; **, p-value < 0,01; ***, p-value < 0,001; n.s., not significant.

The maximum and average daily temperature (°C) and the daily precipitation (mm) were recorded for the entire duration of the experiment. The data produced during the whole period of investigation (July 14 to August 25, 2022) are presented in **Figure 1**, together with Phi2, PhiNO, and PhiNPQ data, which were determined on July 14, 22, and 26 and on August 2, 10, 17, and 25. Despite the hot and dry climates that characterized the entire period, the highest temperatures were remarked in the first part of our time frame (July 14 to August 6). In particular, July 15 and 22 and August 5 showed the highest average and maximum values, respectively, higher than 37°C and 29°C. Contrarily, the highest precipitation levels were observed in correspondence with sudden temperature drops (July 27 and August 18, respectively, 35.8 mm and 75.6 mm).

ANOVA and Tukey’s test on chlorophyll fluorescence parameters were separately conducted for each data collection (**Figure 1**). Interestingly, most of the differences between treatments were observed under the highest temperatures registered (July 14, 22, and 26 and August 2). The data obtained on the hottest days (July 22 and 26) showed significant differences in photosynthetic performance. On both dates, the recorded Phi2 values registered with T3 treatment (0.558 ± 0.065 on July 22 and 0.471 ± 0.141 on July 26) were statically higher than those with Control (0.299 ± 0.033 on July 22 and 0.301 ± 0.031 on July 26).

### 3.3 UHPLC/QTOF-MS metabolic profiling of leaves

Initially, UHPLC/QTOF-MS untargeted metabolomics approach was carried out on grape leaves to obtain a comprehensive view of biochemical plant response to the different biostimulant treatments. Overall, more than 2,400 compounds were putatively annotated and subsequently listed in **Table S3**, together with their respective abundances, composite mass spectra, and retention time.

**Figure 2:**
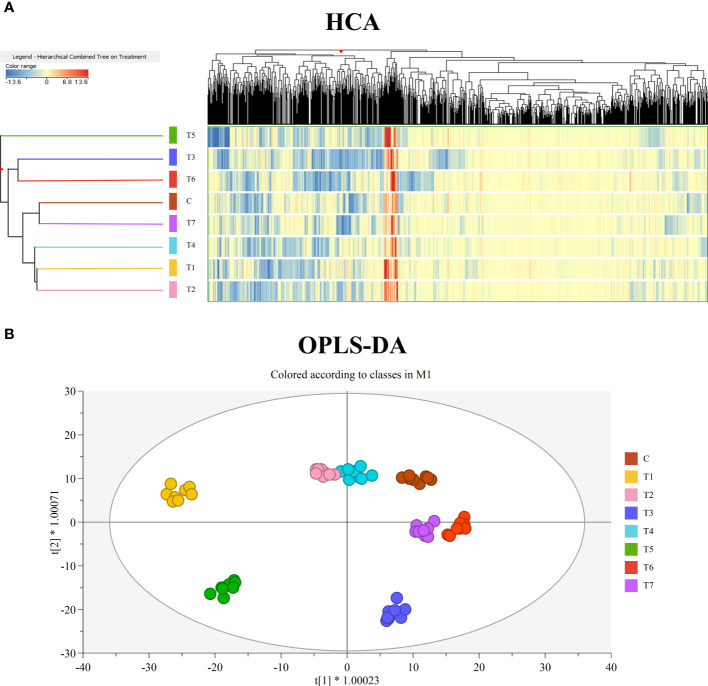
**(A)** Unsupervised hierarchical cluster analysis of grape leaves’ metabolomic profiles, obtained by UHPLC/QTOF-MS untargeted analysis, as a function of the treatment with mycorrhizal-based biostimulants. A fold-change-based heatmap was built, and samples were clustered according to Ward’s algorithm and based on Euclidean distances. **(B)** Orthogonal projections to latent structures discriminant analysis (OPLS-DA) score plot for grape leaves metabolomic following treatment with mycorrhizal-based biostimulants.

First, the fold change-based unsupervised hierarchical cluster analysis was performed to point out clusters on the metabolic signatures of samples (**Figure 2A**). The resulting dendrogram showed two main clusters, the first of which formed by T3 and T6, while the second one by Control, T1, T2, T4, and T7. Within the second cluster, two sub-groups were observed, formed by Control and T7 and by T1, T2, and T4. Interestingly, T5 samples were not merged with any of these clusters, highlighting a different modulation of metabolite profile compared to the other treatments.

Subsequently, unsupervised HCA results were further elaborated by the score plot of the OPLS-DA supervised approach, where sample projection in a two-dimensional space confirmed a clear separation as a function of the treatment factor (**Figure 2B**). The OPLS-DA model was validated by CV-ANOVA (*p*-value = 8E−21) and by inspecting the goodness-of-fit (Q2Y = 0.76) and prediction (R2Y = 0.98) parameters. At the same time, permutation testing (N > 100) excluded model overfitting. Finally, the VIP approach allowed us to select the compounds having the highest discrimination potential (VIP score ≥1.2) in the prediction model. Therein, isoprenoids—mainly diterpenoids, sesquiterpenes, and carotenoids—and alkaloids were mostly represented. The complete list of VIP marker compounds is provided in **Table S4**.

**Figure 3:**
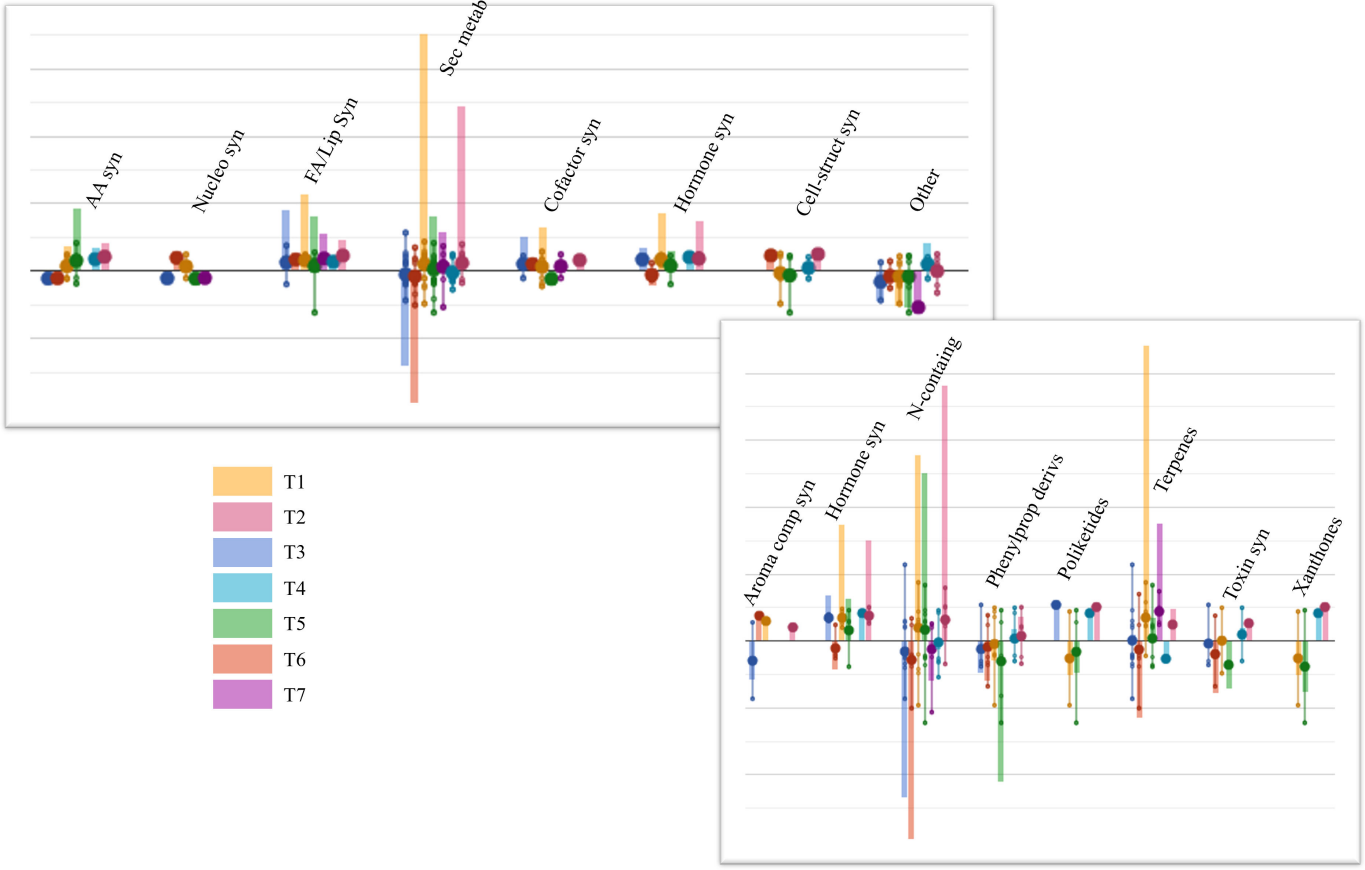
Plant leaves’ metabolic processes **(A)** and the relative details of secondary metabolism **(B)** as affected by arbuscular mycorrhizal fungi (AMF)-based biostimulants compared to Control. The 246 compounds selected by ANOVA (*p*-value ≤ 0.05, Benjamini correction) were subjected to fold change analysis (FC ≥ 1), and the resulting values were loaded into the PlantCyc pathway tool^1^. The x-axis represents each set of metabolic subcategories, while the y-axis corresponds to the cumulative log fold change (FC). The large dots represent the average (mean) of all FCs for the different metabolites in the class, while the small dots represent the individual log FC. AA syn, amino acid biosynthesis; Nucleo syn, nucleoside and nucleotide biosynthesis; FA/Lip syn, fatty acid and lipid biosynthesis; Sec metab, secondary metabolite biosynthesis; Cofactor syn, cofactor, carrier, and vitamin biosynthesis; Cell-struct syn, cell structure biosynthesis; N-containing, nitrogen-containing secondary compound biosynthesis; Phenylprop derivs, phenylpropanoid derivative biosynthesis.

After that, ANOVA (*p*-value ≤ 0.05) allowed us to annotate 246 differential compounds, which appeared significantly affected by the biostimulant treatments, compared to Control (**Table S5**). These compounds were interpreted by the PlantCyc Pathway Tool software as provided in **Figure 3**. Concerning primary metabolism, lipid biosynthesis was mainly impaired by biostimulant application, showing enhanced concentrations of glycolipids and phospholipids in the leaves of each treatment. Notably, this upregulation was observed in T1 and T2, which displayed higher levels of unsaturated lipids. Among these, markedly increased contents of 1-oleoyl-2-palmitoyl-phosphatidylglycerol, 1-palmitoyl-2-linoleoyl-phosphatidylcholine, and 1-18:2-2-18:2-monogalactosyldiacylglycerol were noticed. Moreover, secondary metabolism was also affected by the treatments; in particular, N-containing metabolites, phenylpropanoids, and terpene compounds exhibited the most substantial modulation. Treatments T1, T2, and T5 elicited N-containing metabolites, while T3 and T6 mainly showed an opposite trend. Most of these compounds belonged to alkaloids like sarpagine and norajmaline. The same accumulation trend was observed for terpene biosynthesis, even if the highest accumulation of these metabolites was registered in T1-treated samples. Among others, triterpenoids (3-methyl-1,2-didehydro-2,3-dihydrosqualene, arabidiol, and 11-deoxoglycyrrhetinate), tetraterpenoids (3,4,3′,4′-tetradehydroisozeaxanthin, prolycopene, and canthaxanthin), and sesquiterpenoids were up-modulated. Differently, phenylpropanoid concentrations were markedly decreased by all treatments, except for T2 and T4, which displayed a very slight accumulation of these compounds. Notably, cinnamate, (3*R*,4*R*)-7,2′-dihydroxy-4′-methoxyisoflavanol, 2-hydroxynaringenin, and apigeninidin contents decreased following treatments. Finally, except for the T6 application, a distinctive metabolic reprogramming of the phytohormone profile was achieved in all the treated plants, showing high concentrations of brassinosteroid (6-deoxoteasterone, 3-dehydroteasterone, and campestanol), auxin ((indol-3-yl)acetate), and cytokinin (*trans*-zeatin *N* ^6^-dimethylallyladenine) compounds.

### 3.4 Phenolic profiling of fruits

The untargeted investigation of phenolic profiles in berries highlighted 156 putatively annotated compounds, as provided in the **Supplementary Material** (**Table S6**), with flavonoids and phenolic acids being the most represented (43 and 42 metabolites, respectively). In detail, flavonoids mainly included anthocyanins (cyanidin 3-*O*-galactoside and pelargonidin 3-*O*-rutinoside) and flavonols (quercetin 3-*O*-glucuronide and isorhamnetin 3-*O*-glucoside), while hydroxycinnamic included *p*-coumaroyl glycolic acid and gallic acid 4-*O*-glucoside, and protocatechuic acid 4-*O*-glucoside was the most represented among hydroxybenzoic acids.

Polyphenols were then quantified as phenolic class equivalents, and the resulting values were provided as mg/g (FW). Treatment-specific profiles could be observed; as reported in **Table 2**, significant differences could be observed for all phenolic sub-class equivalents (*p*-value ≤ 0.05), except for flavanols, flavonols, and lignans. In general, small-molecular-weight phenolics and phenolic acids displayed the highest concentrations, ranging from 0.655 mg eq./g to 0.232 mg eq./g and from 0.247 mg eq./g to 0.079 mg eq./g. Overall, T6-treated samples reported the highest values for anthocyanins (0.038 ± 0.005 mg eq./g), phenolic acids (0.214 ± 0.033 mg eq./g), and stilbenes (0.024 ± 0.003 mg eq./g). On the contrary, T5-treated samples showed the highest concentrations of flavanones/flavones/isoflavonoids/dihydroflavonols (0.011 ± 0.002 mg eq./g), flavanols (0.033 ± 0.021 mg eq./g), flavonols (0.025 ± 0.009 mg eq./g), and small-molecular-weight phenolics (0.527 ± 0.001 mg eq./g). Finally, the highest content of lignans (0.073 ± 0.005 mg eq./g) was registered following the T1 application. Among phenolic profiles, it is worth emphasizing the remarkable variance between T6 and T4 for anthocyanins and phenolic acids and between T6 and T5 for flavanones/flavones/dihydrochalcones/isoflavonoids and small-molecular-weight phenolics.

**Table 2:** Semi-quantitative phenolic profile (mg/g^1^ FW equivalents per sub-class), following identification through UHPLC/QTOF-MS untargeted analysis in grape berries following AMF-based biostimulant treatments.

Source of variance	Anthocyanins	Flavanols	Flavanones/ flavones/ isoflavonoids/ dihydroflavonols	Phenolic acids	Flavonols	Lignans	Stilbenes	Other polyphenols
	mg Eq./g DM	mg Eq./g DM	mg Eq./g DM	mg Eq./g DM	mg Eq./g DM	mg Eq./g DM	mg Eq./g DM	mg Eq./g DM
**Control**	0.030 ± 0.002^ab^	0.020 ± 0.016	0.010 ± 0.000^ab^	0.092 ± 0.008^c^	0.020 ± 0.002	0.067 ± 0.010	0.004 ± 0.000^c^	0.444 ± 0.045^abc^
**T1**	0.034 ± 0.002^ab^	0.011 ± 0.009	0.008 ± 0.002^ab^	0.152 ± 0.033^b^	0.014 ± 0.005	0.073 ± 0.005	0.010 ± 0.013^ab^	0.301 ± 0.018^bc^
**T2**	0.026 ± 0.002^ab^	0.018 ± 0.006	0.010 ± 0.002^ab^	0.106 ± 0.013^bc^	0.021 ± 0.006	0.050 ± 0.020	0.003 ± 0.000^c^	0.455 ± 0.103^ab^
**T3**	0.027 ± 0.010^ab^	0.031 ± 0.019	0.010 ± 0.001^ab^	0.106 ± 0.021^bc^	0.021 ± 0.002	0.067 ± 0.010	0.004 ± 0.001^c^	0.438 ± 0.069^abc^
**T4**	0.023 ± 0.001^b^	0.020 ± 0.012	0.009 ± 0.000^ab^	0.096 ± 0.017^c^	0.017 ± 0.005	0.062 ± 0.012	0.004 ± 0.001^bc^	0.377 ± 0.043^abc^
**T5**	0.029 ± 0.003^ab^	0.033 ± 0.021	0.011 ± 0.002^a^	0.109 ± 0.022^bc^	0.025 ± 0.009	0.052 ± 0.014	0.003 ± 0.000^c^	0.527 ± 0.068^a^
**T6**	0.038 ± 0.005^a^	0.009 ± 0.004	0.007 ± 0.001^b^	0.214 ± 0.033^a^	0.015 ± 0.006	0.043 ± 0.018	0.024 ± 0.003^a^	0.278 ± 0.046^c^
**T7**	0.0027 ± 0.008^ab^	0.013 ± 0.006	0.008 ± 0.001^ab^	0.106 ± 0.007^bc^	0.018 ± 0.007	0.050 ± 0.023	0.003 ± 0.000^c^	0.522 ± 0.133^a^
**Significance**	***	n.s.	***	***	n.s.	n.s.	***	***

Data are presented as mean values ± standard deviation (n = 4). Superscript letters within each column indicate homogenous sub-classes resulting from ANOVA (*p-value* < 0.05, Tukey’s post hoc test). AMF, arbuscular mycorrhizal fungi. ***, p-value < 0,001; n.s., not significant.

### 3.5 Fruit quality and quantity traits

Non-significant differences (*p*-value ≤ 0.05) could be observed for yield, pH, titratable acidity, malic acid, and °Brix among treatments (**Table S7**), even if statistical analysis with a less restrictive threshold (*p*-value ≤ 0.10) on this last response profile would not reject the H0 hypothesis, having obtained a *p*-value of 0.09.

Contrarily, weight and tartaric acid values were differently affected after AMF-based biostimulants were applied (*p*-value ≤ 0.05). Concerning tartaric acid, Tukey’s test highlighted remarkable differences between T6 and T3 treatments, respectively revealing the highest (3.725 ± 0.365 g/L) and lowest (2.798 ± 0.329 g/L) average values. Significant differences in weight variables were assessed between T4 (98.608 ± 8.480 g) and T2 (68.823 ± 4.836 g).

**Figure 4:**
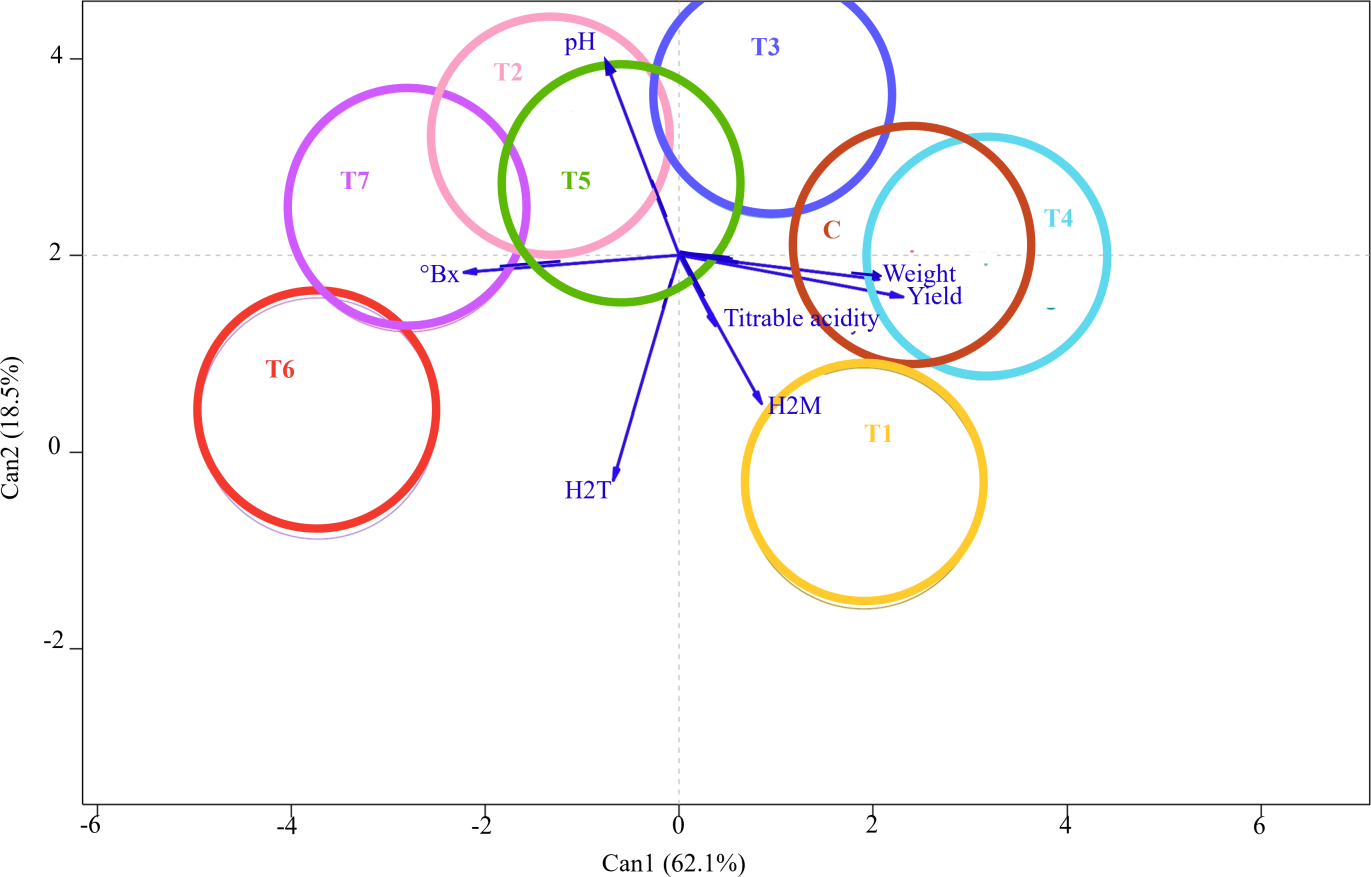
Canonical discriminant analysis (CDA) showing differences in the qualitative and quantitative profiles of grape berries following seven different treatments with arbuscular mycorrhizal fungi (AMF)-based biostimulants (T1, T2, T3, T4, T5, T6, and T7) and Control. The 95% confidence interval of each treatment is shown as a colored circle around the treatment mean. The vectors indicate the contributions of fruit qualitative and quantitative traits to discriminating among treatments; the longer the vector, the stronger its influence in the direction shown.

Nevertheless, MANOVA showed a significant effect on the treatment factor by assessing the multiple dependent variables simultaneously (*p*-value = 9.269e^−05^). Subsequently, CDA was performed to thoroughly examine the discriminant power of the independent variables in distinguishing between treatment groups. CDA graphical output provided a low-dimensional visualization of between-group variation and vectors reflecting the weights of the fruit qualitative and quantitative variables in a canonical space (**Figure 4**). The output showed that a substantial amount of the variance (62%) of the between-treatment mean differences was accounted for by the first canonical variable (Can1). In comparison, the second canonical variable (Can2), orthogonal and uncorrelated to the first one, explained the remaining 18.5%. Eight colored circles, each representing the 95% confidence interval around the treatment mean, were projected on a plane that showed the largest variation between them. The clear separation between these circles indicated that the independent variables significantly contributed to the prediction of grape samples membership to the treatment group and revealed the effective power of the identified discriminant functions for classification and prediction purposes. In contrast, overlapping or closely clustered circles indicated lower discriminant power and potential similarities between treatments. Interestingly, Can1 largely separated Control from T6, T7, and T2, while Can2 mainly differentiated T1 from Control. Contrarily, T4 and Control samples were not well disjoined by canonical variables, thus indicating similar qualitative and quantitative profiles. The seven response variables—pH, °Brix, tartaric and malic acids, titratable acidity, weight, and yield—were represented by vectors, whose angles with Can1 and Can2 reflected their correlations. The relative lengths of these vectors pointed to their contribution to discrimination among treatments. Can1 was positively correlated with weight and yield, but °Brix also separated T7 and T6 from control in the opposite direction. Can2 largely reflected tartaric acid and pH, with T1 and T6 having higher sugar content and lower acidity than T2 and T3.

## 4 Discussion

Earlier studies on grapevine have shown that AMF application can be a reliable approach to vine resilience in wine-producing regions (Malhi et al., 2021; Aguilera et al., 2022). Our experiment corroborated the previous findings, implying a different modulation of photosynthetic energy allocation, physiological activity, and, consequently, fruit biochemistry of grapevine plants in a hyper-arid growing season, following the application of seven mycorrhizal-based commercial products.

Weather data recorded during the 2022 growing season at the test site suggested more stressful conditions for grapevines than the average of the last 30 years^2^. This may explain our measurements obtained on photosynthetic performance. Interestingly, comparing the radiation energy partitioning between treatments, a lower proportion of the incident radiation was allocated toward photochemistry (Phi2) under the highest temperatures. Therefore, a higher proportion of the energy allocated toward heat dissipation (PhiNPQ) and other non-regulatory processes (PhiNO) was observed. Grapevine cultivation for winemaking is highly dependent on climate conditions, and particularly, elevated temperatures and moderate water deficit have been associated with severe damage in plant photosynthetic apparatus due to the inhibition of PSII activity, which is supposed to be the most heat-sensitive physiological system of the grapevine (Camejo et al., 2005; Arias et al., 2022). Previous studies involving chlorophyll fluorescence measurements in grapevine have reported a significant decline of PSII photochemical efficiency in response to temperatures above 35°C, referring to this phenomenon as photoinhibition (Maroco et al., 2002; Zulini et al., 2005; Ju et al., 2018).

In this framework, the data collected on the hottest days indicated the ability of AMF–plant symbiosis ability to enhance the efficiency of excitation energy captured by chloroplasts and increase the PSII photochemical capacity in leaves, showing a general reduction in Phi2 and an increase in PhiNO and PhiNPQ in inoculated plants. Accordingly, extensive research over the last few years has comprehensively investigated the effect of AMF on plant photosynthesis under abiotic stress, showing an overall decrease in photoinhibition following the symbiosis (Mo et al., 2016; He et al., 2017; Zai et al., 2021). A recent meta-analysis has proved that photosynthetic rates and stomatal conductance of salt-stressed C3 and C4 plants are positively influenced by AMF (Chandrasekaran et al., 2019). Nevertheless, only a few studies have analyzed the effect of AMF on the photosynthetic performance of grapevine, and far fewer have featured field experiments. Among these studies, higher photosynthetic rates on AMF-inoculated Crimson grapevines have been pointed out by Nicolás et al. (2015), confirming significant increases in net carbon assimilation (A_N_), water use efficiency (WUE), and stomatal conductance (gs) values with mycorrhizal symbiosis. Comparable to our study, Torres et al. (2021) have explored the photosynthetic response of young Merlot grapevines to *Rhizophagus intraradices*, *Funneliformis mosseae*, *Glomus aggregatum*, and *Glomus etunicatum* inoculation. Interestingly, AMF positively influenced the photosynthetic performance of the inoculated plant, reporting significantly higher values of A_N_ and WUE in leaves.

AMF inoculation improves plant photosynthesis and PSII functioning mainly by enhancing water uptake, stabilizing membrane structure, and upregulating antioxidant metabolism and osmolyte accumulation (Begum et al., 2020). Indeed, substantial evidence has emerged for robust reprogramming of AMF-inoculated plants’ primary and secondary metabolites, implied in plant antioxidant defense systems against stressful conditions (Kaur and Suseela, 2020; Zhao et al., 2022). These findings support our research, where both unsupervised and supervised multivariate statistics results highlighted remarkable changes in the leaf metabolome of mycorrhized plants. Concerning primary metabolism, major changes have been observed for lipids, especially glycolipids and phospholipids, which were increased by AMF treatments. According to recent evidence, lipids are synthesized by plants and transferred to AMF, thus representing an alternative carbon source to sugars (Stacey et al., 2017; Macabuhay et al., 2022). Moreover, they are important players in establishing symbiosis, acting as constituents of the periarbuscular membrane, fungal spores, and vesicles (Kameoka and Gutjahr, 2022). Nevertheless, both glycolipids and phospholipids are plant membrane elements, and in the presence of stress—especially in lack of water—they are subject to hydrolysis following the activation of specific phospholipases (Toumi et al., 2008). Thus, the increase of unsaturated lipids and, consequently, of plant membrane stabilization reported by our study may be related to the pivotal mycorrhizal role in modulating plant stress tolerance. This result is consistent with the recent study of Goddard et al. (2021) where *Rhizophagus irregularis* colonization in grapevine (cv. Gewurztraminer clone 643) has resulted in significantly higher contents in EOTE, linoleic, and linolenic acids in leaves.

Concerning secondary metabolism, AMF inoculation mainly enhanced the accumulation of alkaloids and terpenoids. It is well established that AMF induce changes in both the abundance and the composition of different plant secondary metabolites to mediate plant–AMF interactions and the establishment of symbiosis with root tissues (Kaur and Suseela, 2020). According to our study, increasing trends of both alkaloids and terpenoids have been reported for many AMF-colonized species, including grapevine (Goddard et al., 2021; Amani Machiani et al., 2022). Most alkaloids, frequently recognized as bioactive compounds, act as defense metabolites against plant pathogens and predators, and several findings have stated their involvement in plant response to drought stress tolerance (Jaleel et al., 2007; Guo et al., 2020). Similarly, terpenoids have been related to many essential biological functions in plants, including growth and development, photosynthesis, and defense activity against biotic and abiotic stresses. According to our results, metabolic changes induced by *Rhizoglomus irregularis* inoculation in cv. Gewurztraminer involved higher levels of terpenoids (Goddard et al., 2021). Moreover, it has been reported that *F. mosseae* symbiosis with cv. Cabernet Sauvignon strongly enhanced the monoterpene concentration, increasing up to 113% compared when with the control plant (Velásquez et al., 2020).

Among phytohormones, strong evidence of auxin, cytokinin, and brassinosteroid accumulation emerged from our treatments, confirming their pivotal role in the modulation of AMF symbiosis (Pérez-Torres et al., 2008). Research over the past few years has focused on the involvement of phytohormones in plant–beneficial microorganism interaction, pointing out their implication from the early recognition/colonization events up to the final arbuscular formation and degradation (Cosme and Wurst, 2013; Bitterlich et al., 2018; Liao et al., 2018). Despite the scanty information available about the effect of microbial biostimulant overall phytohormone profile of grapevines (Torres et al., 2018b), Goddard et al. (2021) confirmed higher levels of salicylic and jasmonic acids in the leaves of *Rhizophagus irregularis*-mycorrhized grapevines.

The elicitation of plant secondary metabolism following inoculation of beneficial microorganisms has been found to promote the accumulation of functional compounds in the edible portions, highlighting the potential role of microbial biostimulants in producing high-quality foods (Ganugi et al., 2021a). Grape composition at harvest is a pivotal point in the wine production chain, representing one of the most important factors determining the future quality of the wine (Guidetti et al., 2010). Phenolic compounds, whose composition depends on the cultivar, ripening conditions, agronomic techniques, and winemaking methods strongly contribute to the sensory properties, color, mouth-fell, and antioxidant characteristics of wine (Rodríguez-Delgado et al., 2002; Merkyte et al., 2020). Our study shows that the AMF-based biostimulants exerted a positive effect on phenolic acids, such as caffeic and caftaric acids, and stilbenes like resveratrol, which are recognized for their contribution to wine color stability, protection against oxidation, and their anti-inflammatory/cardioprotective activity (Flamini et al., 2013). This effect was more evident in T1 and T6, both based on *Rhizophagus* ssp. AMF, which showed high contents of these compounds, compared to the control. This confirms the potential use of mycorrhizal fungi in vineyards as an efficient ecological tool to improve the phenolic composition of grapes, in agreement with previous studies on tomato, saffron, and strawberry (Ganugi et al., 2021a). Interestingly, similar studies on *Rhizophagus* ssp.-mycorrhized grapevines have shown enhanced polyphenol synthesis in fruits of cv. Tempranillo, especially regarding anthocyanin content (Torres et al., 2016; Torres et al., 2018b; Torres et al., 2018c).

Curiously, mycorrhization did not significantly improve the levels of anthocyanins in our experiment. This agrees with the study of Gabriele et al. (2016), who exhibited significantly lower levels of anthocyanins in symbiotic wines than in conventional ones. Despite that, a remarkable variation in anthocyanin content could be observed for two treatments (namely T3 and T4), strengthening how important the fungal strain may be in modulating plant secondary metabolites (Avio et al., 2018). Notably, T3 is the only treatment based on *Rhizophagus iranicus*, while the more generic indication for T4 (*Rhizophagus* ssp.) does not allow us to trace the species present in the biostimulant.

However, the reason behind the discrepancies among biostimulant effects may be related, in addition to the inoculum species, to the different rates and times of microbial treatments (Bulgari et al., 2015; Nephali et al., 2020). Timing and dosage are critical determinants, as the plant metabolomics response may differ depending on the growth stage when the inocula are applied, as well as higher doses might trigger a more pronounced response when compared to lower rates.

The application of MANOVA allowed us to obtain a comprehensive visualization of multi-faceted results, highlighting simultaneous variations in chemical and yield components of fruits depending on the treatment product. Interestingly, the berry weight of T2 treatments significantly decreased compared to non-mycorrhized plants (Control), while no statistical difference was observed for the yield component. This can be translated into a higher number of grapes per treated vine but of a smaller size, and vice versa for Control. This confirms the study of Karoglan et al. (2021), where AMF inoculation on cv. Cabernet Sauvignon significantly decreased the berry weight but increased the number of berries per cluster. As shown in the graphic plot, treatments with higher yield values revealed lower TSS, where total soluble solids (mainly glucose and fructose) were more diluted. Nonetheless, all symbiotic grapes exhibited higher TSS than untreated samples. Like our study, the fruit SSC of AMF-treated plants was higher in Crimson, Tempranillo, and Cabernet Sauvignon cultivars (Nicolás et al., 2015; Torres et al., 2016; Karoglan et al., 2021). Indeed, the AMF capacity to promote the uptake of mineral nutrients by roots and stimulate photosynthetic activity, thereby hastening the accumulation of sugars in fruits, has been established (Cao et al., 2021). However, the AMF influence on TSS concentration in grapes varies significantly depending on the grape variety, as elucidated by Antolín et al. (2020). In the aforementioned study, the symbiotic association of grapevines with AMF resulted in a greater accumulation of sugars in fruits of the Grand Noir, Pasera, and Ambrosina varieties. Conversely, no discernible alterations were observed in the Tinto Velasco, Vivadillo, Tinto Velasco, Graciano, and Morate varieties. Similarly, Karagiannidis et al. (2007) discovered no significant disparities in fruit soluble solids between mycorrhized and non-mycorrhized plants, specifically in the cv. Razaki. Additionally, mycorrhizal treatments failed to induce heightened acidity compared to the control, as stated by Torres et al. (2019). pH levels, titratable acidity, and organic acid levels were unaffected in the presence of AMF, according to the same study.

## 5 Conclusion

In conclusion, our study elucidated the crucial role played by AMF-based biostimulant inoculation in sustaining photosynthetic and physiological activities and modulating fruit quality in grapevines under non-optimal growing conditions.

Interestingly, this is the first work comparing the effects of seven different mycorrhizal-based treatments on grapevine response at both plant and fruit levels. Biostimulant-treated vines showed higher levels of plant photosynthesis and PSII functioning, reflecting an overall decrease in photoinhibition following biostimulant symbiosis. In parallel, untargeted metabolomics followed by multivariate statistics highlighted a substantial reprogramming of metabolism (mainly lipids, alkaloids, and terpenoids) in symbiotic plants. Additionally, biostimulant application exhibited significant variation in chemical and yield components, generally displaying improved contents of polyphenols and sugars, when compared to untreated plants. Despite some effects that were already known, it is worth mentioning the specific role played by the different biostimulants in providing plant resilience and in modulating the phytochemical profile of berries. Noteworthy, future investigations are needed to confirm these findings in other cultivars.

## Data availability statement

The original contributions presented in the study are included in the article/**Supplementary files**, further inquiries can be directed to the corresponding author/s.

## Author contributions

PG, TC, MG, AF, and MT: methodology and validation. PG, MG, ES, LZ, MF, and FA: formal analysis and investigation. PG, ES, TC, and GB: software and data curation. PG, ES, and MF: writing—original draft preparation. TC, EP, AF, VT, and LL: writing—review and editing. MT and LL: resources. LL: conceptualization and project administration. All authors contributed to the article and approved the submitted version.

## Footnotes

1
http://www.plantcyc.org

2
www.arpae.it

## References

[B1] Aguilera P. Ortiz N. Becerra N. Turrini A. Gaínza-Cortés F. Silva-Flores P.. (2022). Application of arbuscular mycorrhizal fungi in vineyards: water and biotic stress under a climate change scenario: new challenge for Chilean grapevine crop. Front. Microbiol. 13. doi: 10.3389/fmicb.2022.826571 PMC8934398 35317261

[B2] Aguín O. Mansilla J. P. Vilariño A. Sainz M. J. (2004). Effects of mycorrhizal inoculation on root morphology and nursery production of three grapevine rootstocks. Am. J. Enol Vitic 55, 108–111. doi: 10.5344/ajev.2004.55.1.108

[B3] Amani Machiani M. Javanmard A. Habibi Machiani R. Sadeghpour A. (2022). Arbuscular mycorrhizal fungi and changes in primary and secondary metabolites. Plants 11, 2183. doi: 10.3390/plants11172183 36079565 PMC9460575

[B4] Antolín M. C. Izurdiaga D. Urmeneta L. Pascual I. Irigoyen J. J. Goicoechea N. (2020). Dissimilar responses of ancient grapevines recovered in Navarra (Spain) to arbuscular mycorrhizal symbiosis in terms of berry quality. Agronomy 10, 473. doi: 10.3390/agronomy10040473

[B5] Arias L. A. Berli F. Fontana A. Bottini R. Piccoli P. (2022). Climate change effects on grapevine physiology and biochemistry: benefits and challenges of high altitude as an adaptation strategy. Front. Plant Sci. 13. doi: 10.3389/fpls.2022.835425 PMC9178254 35693157

[B6] Arrizabalaga M. Morales F. Oyarzun M. Delrot S. Gomès E. Irigoyen J. J.. (2018). Tempranillo clones differ in the response of berry sugar and anthocyanin accumulation to elevated temperature. Plant Sci. 267, 74–83. doi: 10.1016/j.plantsci.2017.11.009 29362101

[B7] Arrizabalaga-Arriazu M. Morales F. Irigoyen J. J. Hilbert G. Pascual I. (2020). Growth performance and carbon partitioning of grapevine Tempranillo clones under simulated climate change scenarios: Elevated CO2 and temperature. J. Plant Physiol. 252. doi: 10.1016/j.jplph.2020.153226 32763650

[B8] Avio L. Turrini A. Giovannetti M. Sbrana C. (2018). Designing the ideotype mycorrhizal symbionts for the production of healthy food. Front. Plant Sci. 9. doi: 10.3389/fpls.2018.01089 PMC6102486 30154803

[B9] Backer R. Rokem J. S. Ilangumaran G. Lamont J. Praslickova D. Ricci E.. (2018). Plant growth-promoting rhizobacteria: Context, mechanisms of action, and roadmap to commercialization of biostimulants for sustainable agriculture. Front. Plant Sci. 871. doi: 10.3389/fpls.2018.01473 PMC6206271 30405652

[B10] Baiano A. De Gianni A. Previtali M. A. Del Nobile M. A. Novello V. de Palma L. (2015). Effects of defoliation on quality attributes of Nero di Troia (Vitis vinifera L.) grape and wine. Food Res. Int. 75, 260–269. doi: 10.1016/j.foodres.2015.06.007 28454955

[B11] Basiru S. Hijri M. (2022). Does commercial inoculation promote arbuscular mycorrhizal fungi invasion? Microorganisms 10 (2), 404. doi: 10.3390/microorganisms10020404 35208858 PMC8879836

[B12] Becares A. A. Fernandez A. F. (2017). Microbiome based identification, monitoring and enhancement of fermentation processes and products. Patent no: WO WO2017096385A1. Available at: https://patents.google.com/patent/US20180363031A1/en*.

[B13] Begum N. Ahanger M. A. Zhang L. (2020). AMF inoculation and phosphorus supplementation alleviates drought induced growth and photosynthetic decline in Nicotiana tabacum by up-regulating antioxidant metabolism and osmolyte accumulation. Environ. Exp. Bot. 176, 104088. doi: 10.1016/j.envexpbot.2020.104088

[B14] Bender S. F. Schlaeppi K. Held A. van der Heijden M. G. A. (2019). Establishment success and crop growth effects of an arbuscular mycorrhizal fungus inoculated into Swiss corn fields. Agric. Ecosyst. Environ. 273, 13–24. doi: 10.1016/j.agee.2018.12.003

[B15] Bitterlich M. Rouphael Y. Graefe J. Franken P. (2018). Arbuscular mycorrhizas: a promising component of plant production systems provided favorable conditions for their growth. Front. Plant Sci. 9. doi: 10.3389/fpls.2018.01329 PMC6139337 30250477

[B16] Bondada B. Shutthanandan J. (2012). Understanding differential responses of grapevine (Vitis vinifera L.) leaf and fruit to water stress and recovery following re-watering. Am. J. Plant Sci. 03, 1232–1240. doi: 10.4236/ajps.2012.39149

[B17] Bruisson S. Maillot P. Schellenbaum P. Walter B. Gindro K. Deglène-Benbrahim L. (2016). Arbuscular mycorrhizal symbiosis stimulates key genes of the phenylpropanoid biosynthesis and stilbenoid production in grapevine leaves in response to downy mildew and grey mould infection. Phytochemistry 131, 92–99. doi: 10.1016/j.phytochem.2016.09.002 27623505

[B18] Buffagni V. Ceccarelli A. V. Pii Y. Miras-Moreno B. Rouphael Y. Cardarelli M.. (2021). The modulation of auxin-responsive genes, phytohormone profile, and metabolomic signature in leaves of tomato cuttings is specifically modulated by different protein hydrolysates. Agronomy 11, 1524. doi: 10.3390/agronomy11081524

[B19] Bulgari R. Cocetta G. Trivellini A. Vernieri P. Ferrante A. (2015). Biostimulants and crop responses: a review. Biol. Agric. Horticult.: Int. J. Sustain. Production Syst. 31, 1–17. doi: 10.1080/01448765.2014.964649

[B20] Calicioglu O. Flammini A. Bracco S. Bellù L. Sims R. (2019). The future challenges of food and agriculture: An integrated analysis of trends and solutions. Sustainability (Switzerland) 11, 222. doi: 10.3390/su11010222

[B21] Camejo D. Rodríguez P. Morales M. A. Dell’Amico J. M. Torrecillas A. Alarcón J. J. (2005). High temperature effects on photosynthetic activity of two tomato cultivars with different heat susceptibility. J. Plant Physiol. 162, 281–289. doi: 10.1016/j.jplph.2004.07.014 15832680

[B22] Cameron D. D. Neal A. L. van Wees S. C. M. Ton J. (2013). Mycorrhiza-induced resistance: More than the sum of its parts? Trends Plant Sci. 18, 539–545. doi: 10.1016/j.tplants.2013.06.004 23871659 PMC4194313

[B23] Cao M. A. Wang P. Hashem A. Wirth S. Abd-Allah E. F. Wu Q. S. (2021). Field inoculation of arbuscular mycorrhizal fungi improves fruit quality and root physiological activity of citrus. Agric. (Switzerland) 11, 1297. doi: 10.3390/agriculture11121297

[B24] Chandrasekaran M. Chanratana M. Kim K. Seshadri S. Sa T. (2019). Impact of arbuscular mycorrhizal fungi on photosynthesis, water status, and gas exchange of plants under salt stress–a meta-analysis. Front. Plant Sci. 10. doi: 10.3389/fpls.2019.00457 PMC6476944 31040857

[B25] Cosme M. Wurst S. (2013). Interactions between arbuscular mycorrhizal fungi, rhizobacteria, soil phosphorus and plant cytokinin deficiency change the root morphology, yield and quality of tobacco. Soil Biol. Biochem. 57, 436–443. doi: 10.1016/j.soilbio.2012.09.024

[B26] Derbew B. Y. Mokashi A. N. Patil C. P. Hegde R. V. (2007). Effect of mycorrhizal inoculation at different salinity levels on root colonization, growth and chlorophyll content of different grape rootstocks (*Vitis* spp.). Trop. Agric. Res. Extension 10, 79–82. doi: 10.4038/tare.v10i0.1875

[B27] Edgar R. C. SINTAX: a simple non-Bayesian taxonomy classifier for 16S and ITS sequences. doi: 10.1101/074161

[B28] Edgar R. C. Haas B. J. Clemente J. C. Quince C. Knight R. (2011). UCHIME improves sensitivity and speed of chimera detection. Bioinformatics 27, 2194–2200. doi: 10.1093/bioinformatics/btr381 21700674 PMC3150044

[B29] Flamini R. Mattivi F. De Rosso M. Arapitsas P. Bavaresco L. (2013). Advanced knowledge of three important classes of grape phenolics: Anthocyanins, stilbenes and flavonols. Int. J. Mol. Sci. 14, 19651–19669. doi: 10.3390/ijms141019651 24084717 PMC3821578

[B30] Fraga H. Molitor D. Leolini L. Santos J. A. (2020). What is the impact of heatwaves on European viticulture? A modelling assessment. Appl. Sci. (Switzerland) 10, 3030. doi: 10.3390/app10093030

[B31] Fraga H. Santos J. A. Malheiro A. C. Oliveira A. A. Moutinho-Pereira J. Jones G. V. (2016). Climatic suitability of Portuguese grapevine varieties and climate change adaptation. Int. J. Climatol. 36, 1–12. doi: 10.1002/joc.4325

[B32] Gabriele M. Gerardi C. Longo V. Lucejko J. Degano I. Pucci L.. (2016). The impact of mycorrhizal fungi on Sangiovese red wine production: Phenolic compounds and antioxidant properties. LWT 72, 310–316. doi: 10.1016/j.lwt.2016.04.044

[B33] Gambetta G. A. (2016). Water stress and grape physiology in the context of global climate change. J. Wine Economics 11, 168–180. doi: 10.1017/jwe.2015.16

[B34] Ganugi P. Martinelli E. Lucini L. (2021a). Microbial biostimulants as a sustainable approach to improve the functional quality in plant-based foods: a review. Curr. Opin. Food Sci. 41, 217–223. doi: 10.1016/j.cofs.2021.05.001

[B35] Ganugi P. Masoni A. Pietramellara G. Benedettelli S. (2019). A review of studies from the last twenty years on plant–arbuscular mycorrhizal fungi associations and their uses for wheat crops. Agronomy 9, 840. doi: 10.3390/agronomy9120840

[B36] Ganugi P. Masoni A. Sbrana C. Dell’Acqua M. Pietramellara G. Benedettelli S.. (2021b). Genetic variability assessment of 127 Triticum turgidum L. accessions for mycorrhizal susceptibility-related traits detection. Sci. Rep. 11, 13426. doi: 10.1038/s41598-021-92837-1 34183734 PMC8239029

[B37] Goddard M. L. Belval L. Martin I. R. Roth L. Laloue H. Deglène-Benbrahim L.. (2021). Arbuscular mycorrhizal symbiosis triggers major changes in primary metabolism together with modification of defense responses and signaling in both roots and leaves of vitis vinifera. Front. Plant Sci. 12. doi: 10.3389/fpls.2021.721614 PMC8424087 34512700

[B38] Guidetti R. Beghi R. Bodria L. (2010). Evaluation of grape quality parameters by a simple vis/NIR system. Trans. ASABE 53, 477–484. doi: 10.13031/2013.29556

[B39] Guo X. Xin Z. Yang T. Ma X. Zhang Y. Wang Z.. (2020). Metabolomics response for drought stress tolerance in chinese wheat genotypes (Triticum aestivum). Plants 9, 520. doi: 10.3390/plants9040520 32316652 PMC7238273

[B40] Hawkins C. Ginzburg D. Zhao K. Dwyer W. Xue B. Xu A.. (2021). Plant Metabolic Network 15: A resource of genome-wide metabolism databases for 126 plants and algae. J. Integr. Plant Biol. 63, 1888–1905. doi: 10.1111/jipb.13163 34403192

[B41] He L. Li C. Liu R. (2017). Indirect interactions between arbuscular mycorrhizal fungi and Spodoptera exigua alter photosynthesis and plant endogenous hormones. Mycorrhiza 27, 525–535. doi: 10.1007/s00572-017-0771-2 28424944

[B42] Jaleel C. A. Manivannan P. Sankar B. Kishorekumar A. Gopi R. Somasundaram R.. (2007). Induction of drought stress tolerance by ketoconazole in Catharanthus roseus is mediated by enhanced antioxidant potentials and secondary metabolite accumulation. Colloids Surf B Biointerfaces 60, 201–206. doi: 10.1016/j.colsurfb.2007.06.010 17643970

[B43] Ju Y.l. Yue X.f. Zhao X.f. Zhao H. Fang Y.l. (2018). Physiological, micro-morphological and metabolomic analysis of grapevine (Vitis vinifera L.) leaf of plants under water stress. Plant Physiol. Biochem. 130, 501–510. doi: 10.1016/j.plaphy.2018.07.036 30096685

[B44] Kameoka H. Gutjahr C. (2022). Functions of lipids in development and reproduction of arbuscular mycorrhizal fungi. Plant Cell Physiol. 63, 1356–1365. doi: 10.1093/pcp/pcac113 35894593 PMC9620820

[B45] Karagiannidis N. Nikolaou N. Ipsilantis I. Zioziou E. (2007). Effects of different N fertilizers on the activity of Glomus mosseae and on grapevine nutrition and berry composition. Mycorrhiza 18, 43–50. doi: 10.1007/s00572-007-0153-2 17987325

[B46] Karoglan M. Radić T. Anić M. Andabaka Ž. Stupić D. Tomaz I.. (2021). Mycorrhizal fungi enhance yield and berry chemical composition of in field grown “cabernet sauvignon” grapevines (V. vinifera l.). Agric. (Switzerland) 11, 615. doi: 10.3390/agriculture11070615

[B47] Kaur S. Suseela V. (2020). Unraveling arbuscular mycorrhiza-induced changes in plant primary and secondary metabolome. Metabolites 10, 1–30. doi: 10.3390/metabo10080335 PMC7464697 32824704

[B48] Kramer D. M. Johnson G. Kiirats O. Edwards G. E. (2004). New fluorescence parameters for the determination of Q A redox state and excitation energy fluxes. 79, 209–218. doi: 10.1023/B:PRES.0000015391.99477.0d 16228395

[B49] Krishna H. Singh S. K. Sharma R. R. Khawale R. N. Grover M. Patel V. B. (2005). Biochemical changes in micropropagated grape (Vitis vinifera L.) plantlets due to arbuscular-mycorrhizal fungi (AMF) inoculation during ex vitro acclimatization. Sci. Hortic. 106, 554–567. doi: 10.1016/j.scienta.2005.05.009

[B50] Kuhn N. Guan L. Dai Z. W. Wu B. H. Lauvergeat V. Gomès E.. (2014). Berry ripening: Recently heard through the grapevine. J. Exp. Bot. 65, 4543–4559. doi: 10.1093/jxb/ert395 24285825

[B51] Liao D. Wang S. Cui M. Liu J. Chen A. Xu G. (2018). Phytohormones regulate the development of arbuscular mycorrhizal symbiosis. Int. J. Mol. Sci. 19, 3146. doi: 10.3390/ijms19103146 30322086 PMC6213213

[B52] Linderman R. G. Davis E. A. (2001). Comparative response of selected grapevine rootstocks and cultivars to inoculation with different mycorrhizal fungi. Am. J. Enol. Vitic. 52, 8–11. doi: 10.5344/ajev.2001.52.1.8

[B53] Macabuhay A. Arsova B. Walker R. Johnson A. Watt M. Roessner U. (2022). Modulators or facilitators? Roles of lipids in plant root–microbe interactions. Trends Plant Sci. 27, 180–190. doi: 10.1016/j.tplants.2021.08.004 34620547

[B54] Malhi G. S. Kaur M. Kaushik P. AlYemeni M. N. Alsahli A. A. Ahmad P. (2021). Arbuscular mycorrhiza in combating abiotic stresses in vegetables: An eco-friendly approach. Saudi J. Biol. Sci. 28, 1465–1476. doi: 10.1016/j.sjbs.2020.12.001 33613074 PMC7878692

[B55] Maroco J. P. Rodrigues M. L. Lopes C. Chaves M. M. (2002). Limitations to leaf photosynthesis in field-grown grapevine under drought—metabolic and modelling approaches. Funct. Plant Biol. 29 (4), 451–459. doi: 10.1071/PP01040 32689490

[B56] Matthews M. A. Anderson M. M. (1988). Fruit ripening in vitis vinifera L.: responses to seasonal water deficits. Am. J. Enol. Vitic. 39, 313–320. doi: 10.5344/ajev.1988.39.4.313

[B57] Merkyte V. Longo E. Windisch G. Boselli E. (2020). Phenolic compounds as markers of wine quality and authenticity. Foods 9, 1785. doi: 10.3390/foods9121785 33271877 PMC7760515

[B58] Mo Y. Wang Y. Yang R. Zheng J. Liu C. Li H.. (2016). Regulation of plant growth, photosynthesis, antioxidation and osmosis by an arbuscular mycorrhizal fungus in watermelon seedlings under well-watered and drought conditions. Front. Plant Sci. 7. doi: 10.3389/fpls.2016.00644 PMC4862978 27242845

[B59] Mori K. Goto-Yamamoto N. Kitayama M. Hashizume K. (2007). Loss of anthocyanins in red-wine grape under high temperature. J. Exp. Bot. 58, 1935–1945. doi: 10.1093/jxb/erm055 17452755

[B60] Nephali L. Piater L. A. Dubery I. A. Patterson V. Huyser J. Burgess K.. (2020). Biostimulants for plant growth and mitigation of abiotic stresses: A metabolomics perspective. Metabolites 10 (12), 505. doi: 10.3390/metabo10120505 33321781 PMC7764227

[B61] Nicolás E. Maestre-Valero J. F. Alarcón J. J. Pedrero F. Vicente-Sánchez J. Bernabé A.. (2015). Effectiveness and persistence of arbuscular mycorrhizal fungi on the physiology, nutrient uptake and yield of Crimson seedless grapevine. J. Agric. Sci. 153, 1084–1096. doi: 10.1017/S002185961400080X

[B62] Nikolaou N. Angelopoulos K. Karagiannidis N. (2003). Effects of drought stress on mycorrhizal and non-mycorrhizal cabernet sauvignon grapevine, grafted onto various rootstocks. Exp. Agric. 39, 241–252. doi: 10.1017/S001447970300125X

[B63] Nogales A. Rottier E. Campos C. Victorino G. Costa J. M. Coito J. L.. (2021). The effects of field inoculation of arbuscular mycorrhizal fungi through rye donor plants on grapevine performance and soil properties. Agric. Ecosyst. Environ. 313, 107369. doi: 10.1016/j.agee.2021.107369

[B64] OIV Compendium of International Methods of Wine and Must Analysis (2021). International Organisation of Vine and Wine, 35 Rue de Monceau, 75008 Paris.

[B65] Paz C. Opik M. Bulascoschi L. Bueno C. G. Galetti M. (2021). Dispersal of arbuscular mycorrhizal fungi: evidence and insights for ecological studies. Microb. Ecol. 81, 283–292. doi: 10.1007/s00248-020-01582-x 32920663

[B66] Pérez-Torres C. A. López-Bucio J. Cruz-Ramírez A. Ibarra-Laclette E. Dharmasiri S. Estelle M.. (2008). Phosphate availability alters lateral root development in Arabidopsis by modulating auxin sensitivity via a mechanism involving the TIR1 auxin receptor. Plant Cell 20, 3258–3272. doi: 10.1105/tpc.108.058719 19106375 PMC2630440

[B67] Rodríguez-Delgado M.Á. González-Hernández G. Conde-González J. E. Pérez-Trujillo J. P. (2002). Principal component analysis of the polyphenol content in young red wines. Food Chem. 78 (4), 523–532. doi: 10.1016/S0308-8146(02)00206-6

[B68] Rognes T. Flouri T. Nichols B. Quince C. Mahé F. (2016). VSEARCH: A versatile open source tool for metagenomics. PeerJ 4, e2584. doi: 10.7717/peerj.2584 27781170 PMC5075697

[B69] Rothwell J. A. Perez-Jimenez J. Neveu V. Medina-Remón A. M’Hiri N. García-Lobato P.. (2013). Phenol-Explorer 3.0: A major update of the Phenol-Explorer database to incorporate data on the effects of food processing on polyphenol content. Database 2013, bat070. doi: 10.1093/database/bat070 24103452 PMC3792339

[B70] Rouphael Y. Colla G. (2020). Editorial: biostimulants in agriculture. Front. Plant Sci. 11. doi: 10.3389/fpls.2020.00040 PMC7010726 32117379

[B71] Sadras V. O. Moran M. A. (2013). Nonlinear effects of elevated temperature on grapevine phenology. Agric. For Meteorol. 173, 107–115. doi: 10.1016/j.agrformet.2012.10.003

[B72] Salek R. M. Neumann S. Schober D. Hummel J. Billiau K. Kopka J.. (2015). COordination of Standards in MetabOlomicS (COSMOS): facilitating integrated metabolomics data access. Metabolomics 11, 1587–1597. doi: 10.1007/s11306-015-0810-y 26491418 PMC4605977

[B73] Schymanski E. L. Jeon J. Gulde R. Fenner K. Ruff M. Singer H. P.. (2014). Identifying small molecules via high resolution mass spectrometry: Communicating confidence. Environ. Sci. Technol. 48, 2097–2098. doi: 10.1021/es5002105 24476540

[B74] Stacey G. Keymer A. Pimprikar P. Wewer V. Huber C. Brands M.. (2017). Lipid transfer from plants to arbuscular mycorrhiza fungi. eLife 6, e29107. doi: 10.7554/eLife.29107.001 28726631 PMC5559270

[B75] Tietz S. Hall C. C. Cruz J. A. Kramer D. M. (2017). NPQ(T): a chlorophyll fluorescence parameter for rapid estimation and imaging of non-photochemical quenching of excitons in photosystem-II-associated antenna complexes. Plant Cell Environ. 40, 1243–1255. doi: 10.1111/pce.12924 28699261

[B76] Torres N. Goicoechea N. Morales F. Antolín M. C. (2016). Berry quality and antioxidant properties in Vitis vinifera cv. Tempranillo as affected by clonal variability, mycorrhizal inoculation and temperature. Crop Pasture Sci. 67, 961–977. doi: 10.1071/CP16038

[B77] Torres N. Antolín M. C. Goicoechea N. (2018a). Arbuscular mycorrhizal symbiosis as a promising resource for improving berry quality in grapevines under changing environments. Front. Plant Sci. 9. doi: 10.3389/fpls.2018.00897 PMC6034061 30008729

[B78] Torres N. Goicoechea N. Carmen Antolín M. (2018b). Influence of irrigation strategy and mycorrhizal inoculation on fruit quality in different clones of Tempranillo grown under elevated temperatures. Agric. Water Manag. 202, 285–298. doi: 10.1016/j.agwat.2017.12.004

[B79] Torres N. Goicoechea N. Zamarreño A. M. Carmen Antolín M. (2018c). Mycorrhizal symbiosis affects ABA metabolism during berry ripening in Vitis vinifera L. cv. Tempranillo grown under climate change scenarios. Plant Sci. 274, 383–393. doi: 10.1016/j.plantsci.2018.06.009 30080626

[B80] Torres N. Yu R. Kurtural S. K. (2021). Arbuscular mycrorrhizal fungi inoculation and applied water amounts modulate the response of young grapevines to mild water stress in a hyper-arid season. Front. Plant Sci. 11. doi: 10.3389/fpls.2020.622209 PMC7840569 33519880

[B81] Toumi I. Gargouri M. Nouairi I. Moschou P. N. Ben Salem-Fnayou A. Mliki A.. (2008). Water stress induced changes in the leaf lipid composition of four grapevine genotypes with different drought tolerance. Biologia Plantarum 52, 161–164. doi: 10.1007/s10535-008-0035-2

[B82] Trouvelot S. Bonneau L. Redecker D. Van Tuinen D. Adrian M. Wipf D. (2015). Arbuscular mycorrhiza symbiosis in viticulture: a review. Agron. Sustain. Dev. 35, 1449–1467. doi: 10.1007/s13593-015-0329-7

[B83] Turrini A. Avio L. Giovannetti M. Agnolucci M. (2018). Functional complementarity of arbuscular mycorrhizal fungi and associated microbiota: the challenge of translational research. Front. Plant Sci. 9. doi: 10.3389/fpls.2018.01407 PMC6166391 30319670

[B84] van der Heyde M. Ohsowski B. Abbott L. K. Hart M. (2017). Arbuscular mycorrhizal fungus responses to disturbance are context-dependent. Mycorrhiza 27, 431–440. doi: 10.1007/s00572-016-0759-3 28120111

[B85] Velásquez A. Vega-Celedón P. Fiaschi G. Avio L. Giovannetti M. D’Onofrio C.. (2020). Responses of Vitis vinifera cv. Cabernet Sauvignon roots to the arbuscular mycorrhizal fungus Funneliformis mosseae and the plant growth-promoting rhizobacterium Ensifer meliloti include changes in volatile organic compounds. Mycorrhiza 30, 161–170. doi: 10.1007/s00572-020-00933-3 31974639

[B86] Woo S. L. Pepe O. (2018). Microbial consortia: Promising probiotics as plant biostimulants for sustainable agriculture. Front. Plant Sci. 9. doi: 10.3389/fpls.2018.01801 PMC6288764 30564264

[B87] Yarberry W. (2021). Dplyr. CRAN recipes: DPLYR, stringr, lubridate, and regex in R. 1–58. USA: APRESS.

[B88] Zai X. M. Fan J. J. Hao Z. P. Liu X. M. Zhang W. X. (2021). Effect of co-inoculation with arbuscular mycorrhizal fungi and phosphate solubilizing fungi on nutrient uptake and photosynthesis of beach palm under salt stress environment. Sci. Rep. 11, 5761. doi: 10.1038/s41598-021-84284-9 33707467 PMC7970979

[B89] Zhao Y. Y. Cartabia A. Lalaymia I. Declerck S. (2022). Arbuscular mycorrhizal fungi and production of secondary metabolites in medicinal plants. Mycorrhiza 32, 221–256. doi: 10.1007/s00572-022-01079-0 35556179 PMC9184413

[B90] Zulini L. Rubinigg M. Zorer R. Bertamini M. (2005). Effects of drought stress on chlorophyll fluorescence and photosynthetic pigments in grapevine leaves (*Vitis vinifera* cv.'White Riesling'). Int. Workshop Adv. Grapevine Wine Res. 754, 289–294. doi: 10.17660/ActaHortic.2007.754.37

